# The link between basic visual processing and higher-level social cognition: Eye gaze perception as a bridge in a transdiagnostic sample enriched with social dysfunction

**DOI:** 10.1017/S0033291726104589

**Published:** 2026-05-14

**Authors:** Kelly Mathis, Scott D. Blain, Laura Locarno, Carly A. Lasagna, Serena DeStefani, Jacob Kraft, Costanza Colombi, Katharine N Thakkar, Cynthia Z Burton, Ivy F. Tso

**Affiliations:** 1 Department of Psychiatry and Behavioral Health, The Ohio State University, Columbus, OH, United States; 2 Department of Psychology, University of Michigan, Ann Arbor, MI, United States; 3 Department of Psychology, University of South Dakota, Vermillion, SD, United States; 4 IRCCS Stella Maris Foundation, 56128 Pisa, Italy; 5 Department of Psychology, Michigan State University, East Lansing, MI, United States; 6 Department of Psychiatry, University of Michigan, Ann Arbor, MI, United States

**Keywords:** contrast sensitivity, emotion recognition, gaze perception, social cognition, theory of mind, transdiagnostic, vision, visual integration

## Abstract

**Background:**

Social cognitive deficits are common across many psychiatric conditions and contribute to broader social dysfunction. One hypothesized mechanism involves altered basic visual processing, which may disrupt the perception of low-level social cues and, in turn, compromise broader social cognitive processes. Here, we examined relations between basic visual processing and different levels of social cognition in a transdiagnostic youth sample.

**Methods:**

A sample of 148 youth, ranging from healthy individuals to individuals with neuropsychiatric diagnoses and significant social dysfunction, completed two measures of basic visual processing (contrast sensitivity and visual integration) and a battery of social cognition tasks spanning lower-level (gaze perception) to mid-level (emotion recognition) to higher-level (theory of mind) social cognition. We used a four-level path model to test whether basic visual processing predicts gaze perception, which in turn predicts emotion recognition, which predicts theory of mind.

**Results:**

Poorer contrast sensitivity and visual integration were associated with less precise gaze perception, which was, in turn, associated with worse emotion recognition, which was associated with worse theory of mind. This four-level path model demonstrated good fit and showed superior fit compared to alternative models.

**Conclusions:**

These findings suggest that basic visual processing influences the perception of basic social cues (e.g. gaze direction), which subsequently impairs more complex social perception and inference. Notably, this study extends prior observations from individuals with chronic schizophrenia to a transdiagnostic youth sample, indicating that altered basic visual processing may be a shared mechanism contributing to social cognitive deficits across psychiatric disorders and illness stages.

## Introduction

Social cognition, the set of psychological processes that enable interpretation of social information, is impaired across multiple psychiatric conditions, including schizophrenia, autism spectrum disorder, and social anxiety disorder (Cotter et al., 2018; Green, Horan, & Lee, 2015; Hezel & McNally, 2014). These impairments contribute to significant functional difficulties, including problems in daily interactions, occupational functioning, and maintaining relationships (Bishop-Fitzpatrick, Mazefsky, Eack, & Minshew, 2017; Braak et al., 2022; Fett et al., 2011; Trevisan & Birmingham, 2016). Yet effective treatments remain limited (Haime et al., 2021), underscoring the need to identify underlying mechanisms that could serve as intervention targets. Given that social cognitive deficits span multiple diagnostic categories, transdiagnostic approaches targeting shared mechanisms may offer promise for greater accessibility and efficacy by reducing the need for disorder-specific interventions (Meidlinger & Hope, 2017; Sauer-Zavala et al., 2017).

One candidate mechanism of social impairments is basic visual processing. Both contrast sensitivity, the capacity to discern subtle luminance differences between an object and its background (Kaur & Gurnani, 2024), and visual integration, the ability to unify spatially discrete visual elements into a coherent percept (Grillini, Renken, & Cornelissen, 2019), appear particularly relevant to processing faces, which are ubiquitous sources of social information. Contrast sensitivity aids in distinguishing the iris from the sclera and individual facial features from the broader face; indeed, contrast sensitivity has been linked to facial expression discrimination in healthy individuals (Avidan et al., 2002). Visual integration is necessary to combine these discrete features into a holistic percept (Farah, Wilson, Drain, & Tanaka, 1998), which underlies perception of gaze direction, facial expression, and face identity. Critically, deficits in both contrast sensitivity and visual integration are documented across conditions marked by social dysfunction, including schizophrenia and autism (Behrmann, Thomas, & Humphreys, 2006; Silverstein et al., 2022; Tso, Carp, Taylor, & Deldin, 2014). Moreover, visual processing deficits can emerge early in the illness course and may worsen over time in psychosis (Keane, Paterno, Kastner, & Silverstein, 2016), highlighting the importance of examining these links during earlier stages of psychopathology – when processes are less confounded by illness chronicity or medication effects and when interventions may be most effective.

How might basic visual processing influence broader social cognition? We propose that eye gaze perception serves as a mechanistic bridge. Gaze perception relies on the visual abilities described above: distinguishing the sclera, iris, and pupil requires contrast sensitivity, while integrating these elements with head orientation to determine where someone is looking requires visual integration. Importantly, gaze perception is disrupted across psychiatric disorders with significant social dysfunction, including schizophrenia, autism, and social anxiety (Hooker & Park, 2005; Pantelis & Kennedy, 2017; Schulze, Renneberg, & Lobmaier, 2013). This cross-diagnostic disruption suggests that altered gaze perception may represent a common pathway through which basic visual processing deficits compromise higher-level social cognition.

The link from gaze perception to higher-level social cognition likely operates through social attention and emotion recognition. Gaze direction conveys critical social information – attention, intention, and emotional state (Emery, 2000). Gaze behavior varies systematically with emotion: gaze aversion accompanies shame or anxiety, while direct gaze signals approach-oriented states such as interest or affection. In healthy individuals, direct gaze facilitates perception of approach-oriented emotions (happiness, anger), while averted gaze facilitates perception of avoidance-oriented emotions (sadness, fear; Adams & Kleck, 2005). Thus, difficulty distinguishing direct from averted gaze may impair emotion recognition.

Emotion recognition, in turn, provides scaffolding for theory of mind, the ability to infer others’ mental states, including their thoughts, intentions, and beliefs (Frith & Frith, 2005). The emotions expressed by others serve as informative cues enabling observers to infer underlying intentions; for example, accurately recognizing that someone feels sad rather than angry enables the inference that their stance is one of hurt rather than hostility. Developmental research indicates that emotion recognition emerges prior to theory of mind and may support its development (Chakrabarti & Baron-Cohen, 2006; O’Brien et al., 2011), suggesting that one must first recognize emotions before understanding attitudes and beliefs.

These considerations suggest a hierarchical model in which basic visual processing supports gaze perception, which in turn supports emotion recognition, which supports theory of mind. Although individual links in this chain have been established in prior research (Blain et al., 2023; Itier & Batty, 2009; Lasagna et al., 2020; Sergi, Rassovsky, Nuechterlein, & Green, 2006; Tso, Carp, Taylor, & Deldin, 2014), no study has directly tested an integrated pathway from basic visual processing to higher-level social cognition within a single model. Moreover, prior work has focused primarily on chronic schizophrenia; whether these relationships generalize across psychiatric conditions and to earlier illness stages remains unclear.

### The current study

The present study addresses these gaps by testing an integrated path model linking basic visual processing (contrast sensitivity and visual integration) to higher-level social cognition (theory of mind) through gaze perception and emotion recognition in a transdiagnostic sample of youth. By including both healthy participants and individuals with neuropsychiatric diagnoses and significant social dysfunction, we maximized variability in social cognitive performance while targeting individuals most likely to benefit from intervention. This approach aligns with the National Institute of Mental Health’s Research Domain Criteria (RDoC) framework, which emphasizes dimensional assessment of psychological processes across diagnostic categories (Insel et al., 2010). We hypothesized that basic visual processing would predict gaze perception, which would in turn predict emotion recognition, ultimately predicting theory of mind.

## Methods

### Participants

We recruited a transdiagnostic sample (n = 148; 71% female; 74% white) of people ages 14–30 (*M*: 23.2 *SD*: 4.2) via early psychosis clinics, patient registries, social media campaigns, and community outreach. This was done to maximize variability in social functioning. Our sample included 42 participants with no social dysfunction and 106 psychiatric patients with various degrees of social dysfunction. Social dysfunction was defined by a current score of ≤69 on the Mental Illness Research, Education, and Clinical Center’s Global Assessment of Functioning (MIRECC GAF; Niv, Cohen, Sullivan, & Young, 2007) or a score of ≤6 on the Global Functioning Social Scales (GF Social; Cornblatt et al., 2007). Among the patients, 89 met criteria for one or more of the following disorders: autism spectrum disorder (n = 36; ASD), psychosis spectrum disorder (n = 22, PSD), and social anxiety disorder (n = 64, SAD). The remaining 17 had subclinical traits of these disorders. All diagnoses were confirmed using the Mini International Neuropsychiatric Interview for DSM-5 (MINI; Sheehan & Lecrubier, 2017), except for autism, which was defined by a previous provider diagnosis or a classification of ‘ASD’ on the Autism Diagnostic Observation Schedule Version 2 (Hus & Lord, 2014).

All participants had a full-scale IQ (FSIQ) ≥ 80 (as assessed using the Wechsler Abbreviated Scale of Intelligence, Second Edition; Irby & Floyd, 2013), visual acuity of 20/30 or better with correction as needed (see below for assessment details), and were able and willing to provide informed consent (or assent alongside parent consent for minors). Participants were excluded if they had significant neurological abnormalities (i.e. mass lesions, seizure disorder, etc.), a Mendelian disorder (i.e. cystic fibrosis, sickle cell anemia, etc.), or if they had active substance abuse within 30 days prior to data collection, as determined by the MINI (Sheehan & Lecrubier, 2017). See Table 1 for detailed descriptive statistics of demographic information.

**Table 1.** Participant characteristics

Sample characteristics	*n*	*%*	*M*	*SD*	*min*	*max*
Sex						
Male	43	29.05				
Female	105	70.95				
Age			23.24	4.77	14	30
Race						
Asian	26	17.56				
Black	8	5.41				
White	109	73.65				
More than one race	5	3.38				
Ethnicity						
Hispanic	9	6.08				
Non-Hispanic	138	93.24				
Did not answer	1	0.68				
Education (years)^a^			15.05	2.99	8	23
Socio-economic score^b^			2.53	0.89	1	5
FSIQ			114.48	12.07	80	144
Diagnostic group^c^						
PSD only	11	5.47				
ASD only	13	9.38				
SAD only	33	25.78				
Comorbid	32	19.53				
Subclinical	17	10.16				
No psychiatric condition	42	29.69				
Psychotropic medication						
Antipsychotic	22	14.86				
Mood stabilizer	18	12.16				
Antidepressant	58	39.20				
Benzodiazepine	15	10.14				
Stimulant	32	21.62				
Anticholinergic	5	3.38				
Social functioning						
MIRECC GAF Social^d^			69.08	15.71	32	98
GF Social^e^			6.71	1.67	3	10

a Note that some participants are currently in school; their years of education were indexed by the most recent year completed.

b Socio-economic score was assessed using a 5-point Likert scale (1 = highest SES; 5 = lowest SES), derived from parental education and occupational status (Erola, Jalonen, & Lehti, 2016).

c PSD = psychosis spectrum disorder; ASD = autism spectrum disorder; SAD = social anxiety disorder; comorbid = two or more disorders; subclinical = no diagnosis of PSD, ASD, or SAD, but had subclinical traits of these disorders and significant social dysfunction.

d MIRECC GAF = Mental Illness Research, Education, and Clinical Center’s Global Assessment of Functioning (Niv, Cohen, Sullivan, & Young, 2007).

e GF = The Global Functioning Scales (Cornblatt et al., 2007).

### Measures and materials

#### Visual acuity

To ensure that the relationship between basic visual processing and social cognition was not due to individual differences in visual acuity, participants completed the Stanford Acuity Test (StAT; Piech et al., 2020). During the StAT, participants determined the direction of the letter ‘E’ at various sizes. Participants completed the task binocularly (with vision correction if needed) to maintain consistency with other measures in the battery. Visual acuity was expressed using decimal notation, where higher numbers indicated worse vision (i.e. 20/20 and 20/10 correspond to 1 and 0.5 in decimal notation, respectively).

#### Contrast sensitivity

**Figure 1.**
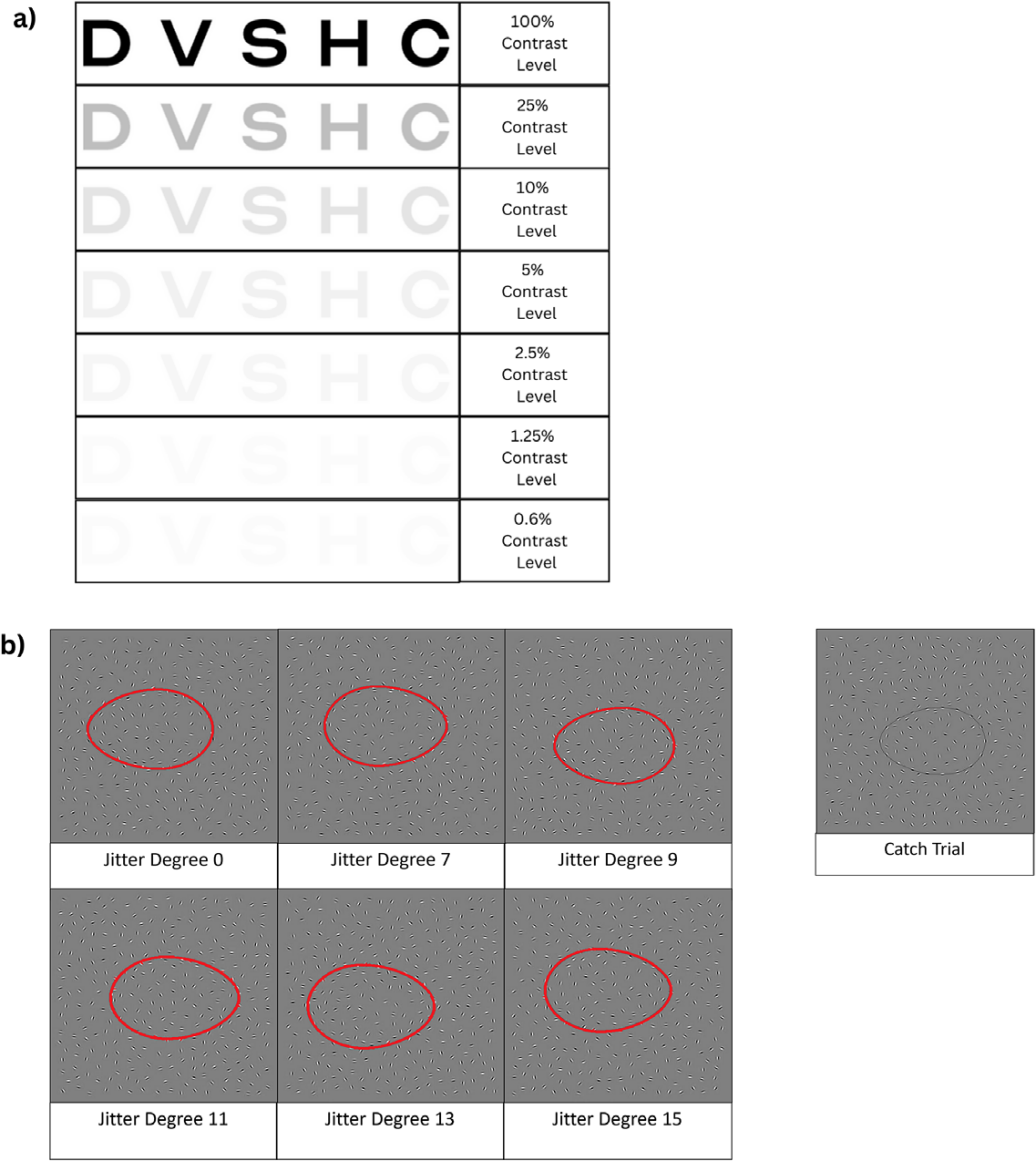
Sample stimuli of basic visual processing tasks. (a) SLOAN Contrast Sensitivity Task: Participants read seven charts presented at decreasing contrast levels (100%, 25%, 10%, 5%, 2.5%, 1.25%, and 0.6%). Each chart was comprised of 60 SLOAN letters, presented in 12 lines in descending font size (only one line is shown here) (b) JOVI: Participants identified the direction (left or right) of the pointy end of an egg-shaped contour, composed of unlinked Gabor elements (highlighted by the red line in the figure; outlines are not present in the actual stimuli), in a field of distractor Gabor elements. Difficulty level increases as the jitter angles of the contour-forming Gabor elements increase. Twenty-four catch trials, where the egg shape was explicitly outlined, were distributed across the task to assess participants’ attention.

Participants read seven letter charts from Precision Vision’s Sloan Low Contrast Letter Set Book (Precision Vision, Woodstock, Illinois, United States of America, n.d.) at varying contrast levels (100%, 25%, 10%, 5%, 2.5%, 1.25%, and 0.6%) from two meters away (Figure 1a). Each chart had 12 lines of five letters, with the lines decreasing in size by .1 log units. The charts were presented in decreasing order of contrast, and participants moved on to the next chart if they misread an entire line.

Each chart was scored using the Logarithm of the Minimum Angle of Resolution (logMAR) notation, based on the number of letters read correctly (Elliott, 2016). LogMAR indexes visual loss, with lower scores indicating better performance (e.g. zero corresponds to Snellen 20/20). Because the 0.6% contrast chart was too difficult for most participants (79% of participants were unable to complete more than two lines), the 1.25% contrast was used as the lowest contrast level in the analysis. Contrast sensitivity was then defined as the difference between participants’ logMAR score on the 1.25% chart (reflecting the lowest usable contrast level) and the 100% chart (reflecting basic visual acuity) (Balcer et al., 2003). Higher difference scores indicate worse contrast sensitivity as more vision was ‘lost’ between the high contrast and the low contrast charts.

#### Visual integration

Participants completed the Jitter Orientation Visual Integration Task (JOVI) by identifying the direction of an egg-shaped object composed of unlinked Gabor elements (Gaussian-modulated sinusoidal luminance distributions) embedded in a field of distractor Gabor elements (Silverstein et al., 2015; Figure 1b). The orientation of the Gabor elements making up the egg-shaped object was shifted at six different jitter angles (0, 7, 9, 11, 13, 15), with a higher angle indicating higher difficulty. Catch trials (trials with the egg shape outlined) served as attention checks; participants with catch trial accuracy <90% were excluded. The task was broken up into two blocks, with a break in between each block. One hundred sixty-eight stimuli were presented: 14 trials per block (12 real trials and 2 catch trials) × 2 blocks × 6 jitter angles. Within each block, trials were divided by difficulty level and presented in increasing order of difficulty, where first all trials at jitter angle 0 were presented, followed by all trials at jitter angle 7, and so on. Each stimulus was preceded by a fixation cross, which stayed on the screen until gaze was maintained there for 250 ms. Following the fixation cross, the stimulus was presented for 2 s. Performance on this task was assessed by the proportion of correct responses on non-catch trials (Strauss et al., 2014).

#### Eye gaze perception

**Figure 2.**
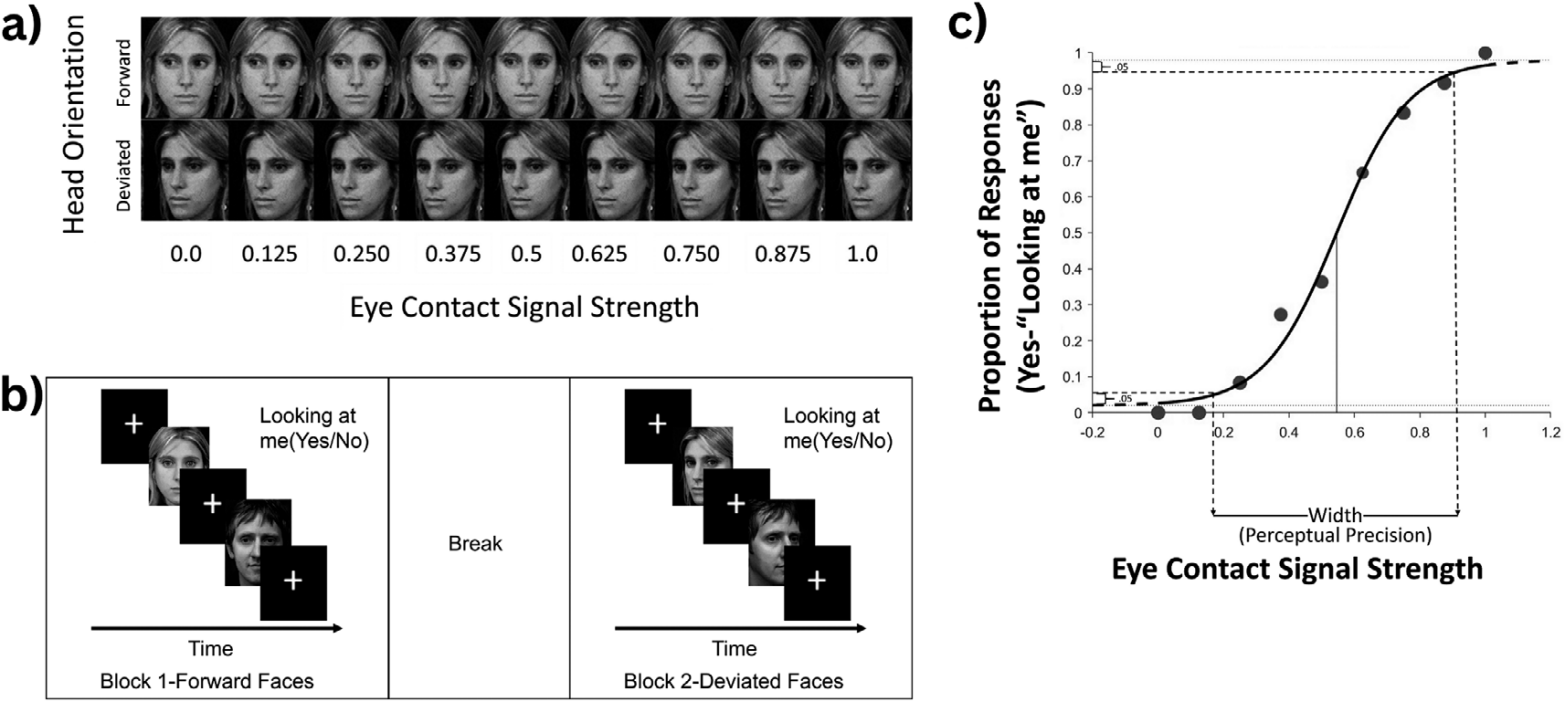
Eye gaze perception task. (a) Participants were presented with faces at nine discrete eye-contact signal strengths, ranging from completely averted (0.0) to completely direct (1.0), at two head orientations (forward and deviated). (b) The task was broken up into two blocks, divided by head orientation, with a break in between. Each block contained 108 faces presented for 2 seconds preceded by a fixation cross. Participants were asked to decide if the person was looking at them by selecting yes or no. (c) A logistic curve was fit to performance data. Perceptual precision was derived from the width of the curve, which was defined as the difference in eye contact signal strength between 5% endorsement and 95% endorsement rate. The function depicted is schematic/illustrative and does not include real participant data.

Participants completed a psychophysical eye gaze perception task (Lasagna et al., 2020), during which eye movements were recorded using SR Research’s Eyelink 1000 eye-tracking camera (SR Research, Ottawa, Ontario, Canada, n.d.; Figure 2).

*Stimuli.* Participants were presented with black and white face images (George, Driver, & Dolan, 2001) at two head orientations (forward facing the center and 30°deviated away from the center) with gaze averted leftward and rightward in various degrees (Figure 2a). The degree of gaze aversion eye contact with the participant, referred to as ‘eye-contact signal strength,’ ranged from 0 (completely averted gaze) to 1 (completely direct gaze) in increments of 0.125. The procedure to generate stimuli at these various eye-contact signal strengths is described in Gu et al. (2024).

*Procedure.* Participants’ eye-to-camera distance was maintained at 55–60 cm throughout the task. Participants were asked to determine if the person in each image was looking at them, via button press (‘yes’ = 1; ‘no’ = 2), based on their first impression. This task consisted of 216 trials (6 actors: 3 male, 3 female) × 9 gaze angles × 2 head orientations (forward, deviated) × 2 gaze directions (eyes averted: leftward, rightward) lasting 2 s each, with a fixation cross in between trials. The face image of the next trial did not appear until gaze was maintained there for 250 ms to ensure that they were paying attention. If no response was made within 2 s of stimulus presentation, the trial was repeated until a response was made, to ensure that all trials were attended to and responded to. The task was divided into two blocks of 108 trials each, grouped by head orientation, with a break in between (Figure 2b).

*Data processing.* We focused on perceptual precision, the ability to discriminate small differences in gaze direction, because this metric reflects the fidelity of low-level sensory encoding rather than response bias or decisional tendencies. Perceptual precision thus captures an intermediate processing stage, positioned between basic visual abilities and higher-order social cognitive outcomes. To derive this measure, for each participant, a psychometric curve was fit to direct gaze endorsement rates against eye-contact signal strength using Bayesian estimation (psignifit 4; Schütt, Harmeling, Macke, & Wichmann, 2016), separately for forward and deviated faces. Leftward and rightward gaze directions were collapsed within signal strengths, resulting in 12 stimulus presentations per signal strength within each head orientation condition. The width of this perception curve, defined as the difference between eye contact signal strength at 5% and 95% endorsement, was used to index ‘perceptual precision’ during gaze perception, with higher values indicating lower precision (Figure 2c). Additional details of the mathematical estimation of this metric are provided in the supplementary material Section 1.1.

*Eye-tracking data.* To ensure that variance in gaze perception was not attributable to differences in attention, participants’ eye movements during the task were analyzed. Dwell time, defined as the total time a participant’s gaze remained within the eye region of the stimuli prior to making a response, was averaged across trials for each participant to index attentional engagement and used as a covariate in follow-up statistical tests (described below). More details on the eye tracking procedure and data processing are in the Supplementary Section 1.2.

### Emotion recognition

Participants completed the Reading the Mind in the Eyes task (RME; Baron-Cohen et al., 2001) to measure emotion recognition. Participants viewed 36 images showing only the eye region of actors, depicting various emotional states. Each image was presented with four emotional adjectives (i.e. arrogant, confused, upset, and relaxed). A definition of each emotional adjective would appear when the cursor is hovered over the word. This design was to reduce the effort of looking up the definition of unknown adjectives, thereby reducing the effect of vocabulary on performance. Participants were asked to select the adjective that best described the emotion depicted in the image from the four options. Overall performance was indexed by the total number of emotions identified correctly.

### Theory of mind

The Awareness of Social Inference Test-Short (TASIT; McDonald et al., 2006) was used to measure theory of mind. Participants viewed 18 videos (15–60 seconds) of social interactions between two or more actors. Following each video, participants answered four questions related to the actors’ thoughts, feelings, statements, and actions. Response options were ‘yes’ or ‘no’ and ‘I don’t know.’ Participants were instructed to respond ‘yes’ or ‘no’ to the best of their ability, and ‘I don’t know’ responses were coded as incorrect. Performance was indexed as the proportion of correct responses across all questions.

### Statistical analyses

Analyses were conducted using R (v4.3.1) and Mplus 8.8 (Muthén & Muthén, 1998). Zero-order Pearson correlations were used to assess bivariate relationships among basic visual processing, gaze perception, and social cognition variables. Then, structural equation modeling was used to test the hypothesized four-level path model (Model A): basic visual processing (contrast sensitivity, visual integration) → gaze perception → emotion recognition → theory of mind. Residual covariances among basic visual processing variables were freely estimated. This sequential ordering reflects developmental and theoretical accounts positioning emotion recognition as intermediate between low-level gaze perception and higher-level, contextually embedded theory of mind. Model fit was evaluated using CFI (≥0.95), TLI (≥0.90), RMSEA (≤0.06), and chi-square.

Alternative models were specified to test the necessity and directionality of the structural path linking emotion recognition and theory of mind. The first alternative model (Model B) removes this path, treating both variables as conditionally independent given gaze perception. The second alternative model is a reversed model (Model C), where gaze perception predicts theory of mind (instead of emotion recognition), which in turn predicts emotion recognition. This allowed for the evaluation of competing directional constraints while preserving the remainder of the model structure. These alternative models were selected to test specific structural assumptions of our hypothesized model (Model A), rather than to exhaustively evaluate all possible permutations.

Finally, hierarchical regression analyses examined whether relationships from our four-level path model remained significant when controlling for demographic and cognitive covariates (age, SES, FSIQ, visual acuity, and dwell time during the gaze task).

## Results

### Bivariate correlations

**Figure 3.**
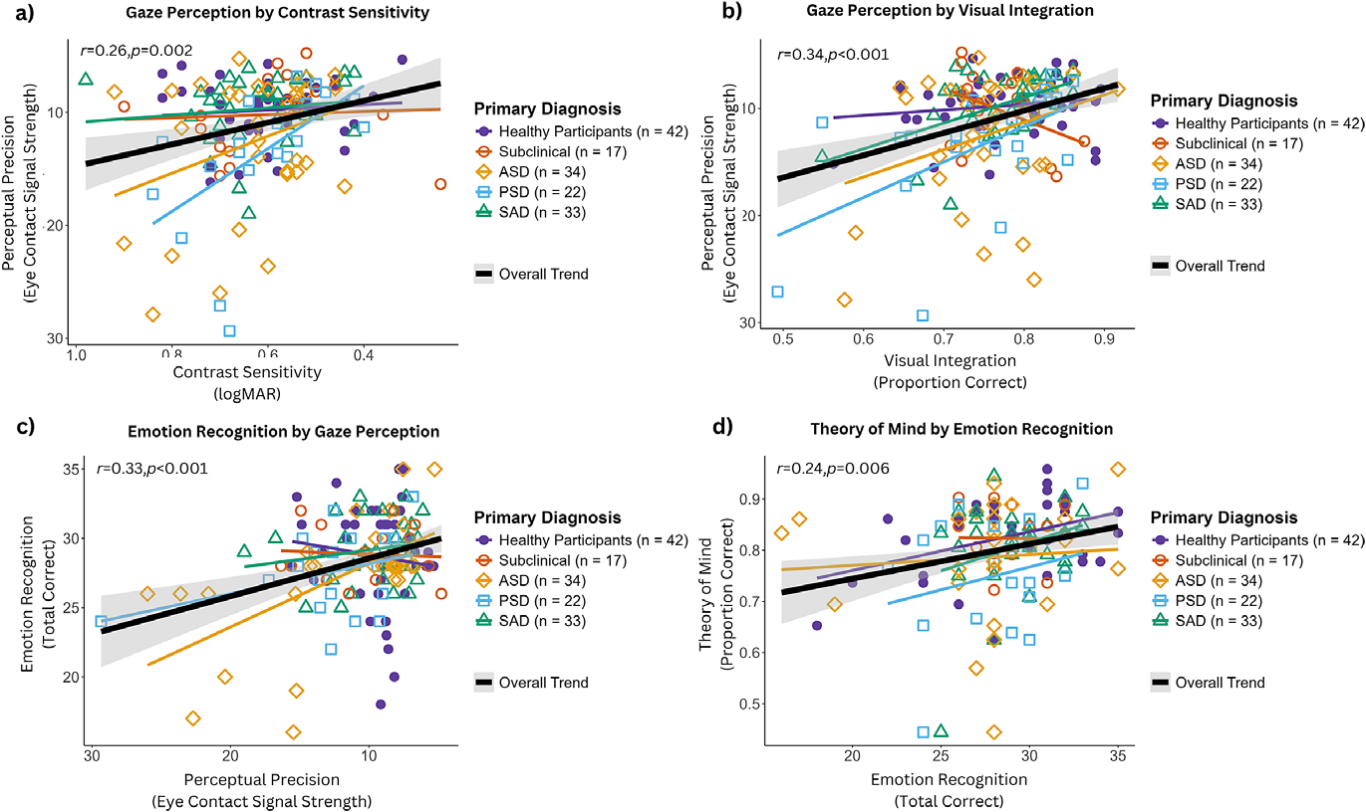
Correlational relationships between visual processing, gaze perception, and social cognition variables. Both visual processing variables showed significant associations with gaze perception. Gaze perception was significantly associated with emotion recognition, which, in turn, was significantly associated with theory of mind. The direction of these associations was consistent across the three diagnostic groups (ASD, PSD, and SAD). Participants who met criteria for multiple conditions were assigned to the group judged to have the greatest clinical impact. *Note*: The perceptual precision axis was inverted in panels a, b, and c because perceptual precision reflects the width of the gaze performance curve, with larger values indicating lower precision. The contrast sensitivity axis was inverted in panel a because contrast sensitivity is expressed in logMAR units, where higher values indicate poorer sensitivity. ASD = autism spectrum disorder; PSD = psychosis spectrum disorder; SAD = social anxiety disorder.

Both basic visual processing variables were significantly associated with gaze perception, and gaze perception was associated with emotion recognition, which was, in turn, associated with theory of mind (Figure 3). Notably, basic visual processing variables were not directly associated with emotion recognition or theory of mind, consistent with an indirect pathway through gaze perception. Full correlation results are provided in the Supplementary Section 2.

Given that separate analyses by clinical subgroup would be severely underpowered, we examined potential diagnosis-specific associations by visualizing the data points in Figure 3. The direction of the associations was consistent across the three clinical diagnostic groups (ASD, PSD, and SAD).

### Path analysis

**Figure 4.**
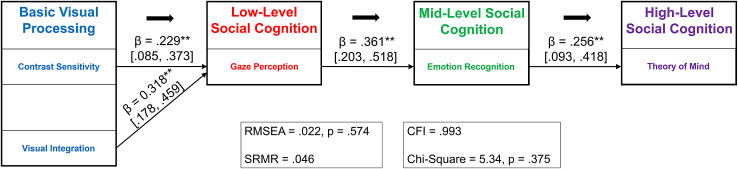
Path model of gaze perception as the link between basic visual processing and broader social cognition (Model A). The hypothesized model demonstrated good fit to the data. Both basic visual processing variables (in blue) were significant predictors of gaze perception (in red), which significantly predicted emotion recognition (in green), which significantly predicted theory of mind (in purple). *Note*: ***p < .001; **p < .01; *p < .05. All path coefficients are reported as absolute values representing associations between better performance on each measure. Gaze perception was indexed by psychophysical width, where higher values indicate worse precision; thus, original coefficients for paths involving gaze perception were negative for visual integration (*β* = −0.32) and for the gaze-to-emotion recognition path (*β* = −0.36).

The hypothesized four-level path model (Model A) demonstrated good fit (χ^2^(5) = 5.34, p = .38; CFI = 0.99; TLI = 0.99; RMSEA = 0.022, 90% CI [0.00, 0.12]; SRMR = 0.046). As shown in Figure 4, both basic visual processing variables significantly predicted gaze perception (contrast sensitivity: *β* = 0.23, p = .002; visual integration: *β* = 0.32, p < .001). Gaze perception predicted emotion recognition (*β* = 0.36, p < .001), and emotion recognition predicted theory of mind (*β* = 0.26, p = .002).

### Model comparison

Model comparisons supported our hypothesized model (Model A) compared with the two alternative models (Model B and Model C). Model B, in which gaze perception predicted both emotion recognition and theory of mind directly, fit adequately (χ^2^(4) = 5.14, p = .27; CFI = 0.98; TLI = 0.95; RMSEA = 0.044). However, the added direct path from gaze perception to theory of mind did not significantly improve model fit (Δχ^2^ = 0.20, Δdf = 1, p = .65) relative to Model A, and the path itself was non-significant. Therefore, based on the principle of parsimony, we selected Model A as the winning model, in which effects propagate sequentially from gaze perception to theory of mind through emotion recognition.

Model C, which reversed the positions of emotion recognition and theory of mind, demonstrated a poor fit (χ^2^(5) = 18.98, p = .002; CFI = 0.71; TLI = 0.47; RMSEA = 0.137). This confirms that gaze perception shares more variance with emotion recognition than with theory of mind. As such, we once again selected Model A as the winning model. Full results of these alternative models are in the Supplementary Section 3.

### Sensitivity analyses

Hierarchical regressions confirmed that all pathways remained significant when controlling for age, SES, full-scale IQ, visual acuity, and eye dwell time (Supplementary Tables S2.1–S2.3).

## Discussion

Our current study set out to characterize the pathway through which basic visual processing impacts higher-level social cognition in a transdiagnostic sample. We hypothesized that basic visual processing would predict higher-level social cognition through gaze perception and emotion recognition. Our findings supported this hierarchical model: contrast sensitivity and visual integration predicted more precise gaze perception, which in turn predicted better emotion recognition, which predicted better theory of mind. Notably, basic visual processing was not directly associated with emotion recognition or theory of mind, consistent with gaze perception serving as an intermediary link. These findings extend prior work in chronic schizophrenia (Sergi et al., 2006; Tso et al., 2014) to a transdiagnostic youth sample.

The observed relationship between basic visual processing and gaze perception aligns with mechanistic accounts of how visual information is extracted from faces. Contrast sensitivity likely facilitates gaze perception by enabling the detection of subtle luminance differences between the sclera, iris, and pupil; visual integration enables the combination of these discrete features into a coherent representation of eye direction. These findings converge with experimental evidence that degraded sensory input impairs gaze perception accuracy (Gu et al., 2024). Critically, these relationships emerged in a transdiagnostic sample, suggesting that altered basic visual processing may represent a shared mechanism contributing to gaze perception difficulties, with potential downstream consequences for everyday social functioning.

Gaze perception, in turn, was associated with emotion recognition, a link that may reflect the tight coupling between gaze direction and emotional expression. Gaze direction varies systematically with affective state: direct gaze accompanies approach-oriented emotions, while averted gaze signals avoidance-oriented states (Adams & Kleck, 2005; Hietanen, 2018). This coupling is particularly relevant to our emotion recognition measure, the RME, which requires participants to infer mental states solely from the eye region. The association between emotion recognition and theory of mind aligns with developmental accounts positioning emotion understanding as foundational to mentalizing (Chakrabarti & Baron-Cohen, 2006). Together, these findings support a processing hierarchy in which accurate perception of low-level visual features supports the extraction of increasingly complex social information.

Model comparison analyses provided additional support for the proposed ordering. An alternative model reversing the positions of emotion recognition and theory of mind demonstrated poor fit, suggesting that emotion recognition shares more variance with gaze perception than does theory of mind. This pattern is also consistent with the perceptual demands of the tasks: the RME requires extracting information from static photographs of eyes, precisely the visual features that gaze perception depends on, whereas the TASIT requires integrating dynamic multimodal cues, including facial expressions, body language, tone of voice, and verbal content. The poor fit of the reversed model thus supports a hierarchical organization in which perceptually grounded processes scaffold more integrative, context-dependent social inference.

These findings have translational implications. If basic visual processing shapes downstream social cognition, then interventions targeting visual processing could potentially yield improvements across the social cognitive hierarchy. Several approaches have shown promise for enhancing visual processing, including vision therapy (Balci & Yalcin, 2013), neural stimulation (Richard, Johnson, Thompson, & Hansen, 2015), adaptive optics correction (De Gracia, Marcos, Mathur, & Atchison, 2011), and perceptual learning (Donato et al., 2024). Notably, one study found that visual remediation therapy improved both visual functioning and positive symptoms in individuals with schizophrenia (Bergson et al., 2024). Taken together with the present transdiagnostic findings, this raises the possibility that visual-focused interventions could confer downstream benefits for social cognition, a hypothesis that warrants further investigation. The transdiagnostic nature of our findings is particularly relevant here: if basic visual processing represents a shared mechanism across conditions, then visual interventions could offer broader applicability than disorder-specific approaches (Martin, Murray, Darnell, & Dorsey, 2018). Furthermore, establishing these connections in adolescents and young adults highlights potential for early intervention during a critical developmental window – when perceptual processes may be more malleable prior to chronic illness.

### Limitations and future directions

Several limitations should be noted. First, according to simulation work, correlations tend to stabilize at substantially larger sample sizes, often around N ≈ 250 (Schönbrodt & Perugini, 2013). Because our sample is more modest, the point estimates reported here may be less stable and more susceptible to sampling variability, resulting in potential effect-size inflation. Future research should replicate these findings in larger samples to assess their robustness. Second, although the transdiagnostic design was intentional for examining shared mechanisms across diagnostic groups, it does not allow the examination of potential diagnosis-specific mechanisms. In this study, subgroup sizes were too small to support formal moderation analyses, leaving open the possibility that diagnosis-specific mechanisms exist. However, visualizations of the results suggest that the three clinical groups (autism, psychosis, and social anxiety disorder) generally showed similar patterns across the associations examined in this study, suggesting that no single diagnosis disproportionately influenced the overall model. However, it remains to be investigated if social cognitive impairments, while present across diagnostic categories, may arise from different underlying mechanisms. For example, some groups may under-attribute mental states, whereas others may over-attribute them. These were not specifically measured in this study. Future studies with larger and more diagnostically homogeneous samples, incorporated with more nuanced and mechanistically comprehensive constructs, will be better positioned to test group-specific pathways. Third, although the proposed ordering of the pathways is theoretically motivated and developmentally plausible, the present cross-sectional analyses test statistical dependencies rather than causal direction; longitudinal or experimental designs would be needed to establish temporal precedence. Fourth, our emotion recognition assessment, the Reading the Mind in the Eyes (RME), has been critiqued for its construct validity, particularly regarding whether it truly indexes emotion recognition or instead taps broader mentalizing abilities (Oakley, Brewer, Bird, & Catmur, 2016). As a result of this ambiguity, paths involving the RME in our model may not reflect emotion recognition in isolation, but instead a combination of emotion recognition and broader social inference processes. A more comprehensive battery that includes measures with stronger construct validity for emotion recognition would allow for a clearer characterization of how basic visual processing relates to different levels of social cognition. Finally, we did not examine how these findings translate to real-world social functioning, underscoring the need for ecologically valid outcome measures in future research.

## Conclusion

Our results demonstrate that basic visual processes are linked to higher-level social cognition through gaze perception and emotion recognition in a transdiagnostic sample. Given the prevalence of social cognitive deficits across psychiatric conditions, identifying upstream perceptual processes that shape downstream social cognitive outcomes may inform the development of more broadly applicable intervention strategies.

## Supporting information

10.1017/S0033291726104589.sm001 Mathis et al. supplementary material Mathis et al. supplementary material

## Supplementary material

The supplementary material for this article can be found at http://doi.org/10.1017/S0033291726104589.
