# Supplementary material for: The link between basic visual processing and higher-level social cognition: Eye gaze perception as a bridge in a transdiagnostic sample enriched with social dysfunction
Source: Psychol Med. 2026 May 14;56:e152. doi: 10.1017/S0033291726104589 (PMC13200143; doi:10.1017/S0033291726104589)
Supplement: Mathis et al. supplementary material [file S0033291726104589sup001.docx]

**Supplementary Materials**

**1 Gaze Task Data Processing Supplemental Information**

*1.1 Gaze Task Psychometric Curve Fitting*

To accommodate differences in perceptual sensitivity for processing gaze of forward and deviated faces, the gaze angle range for the two head orientation conditions was different. Specifically, the forward faces had gaze angles from 0° to 10° in increments of 1.25° and deviated faces had gaze angles from 0° to 24° in increments of 3°. We used a larger range of gaze angles for deviated faces because, in our pilot testing, using the 0° - 10° range resulted in >50% endorsement for even the most averted gaze (10°); this dramatically shifted the perception curve (described below). Using the 0° - 24° for deviated faces results in more centered data, and therefore, more valid estimations of gaze perception metrics.

We used the psignfit 4 toolbox (Schütt et al., 2016) implemented in MATLAB to estimate perceptual precision during gaze perception. To enable comparable mathematical operations between the two head orientation conditions, gaze angles were first converted to a scale of “eye-contact signal strength” ranging from 0 (completely averted) to 1 (completely direct) in increments of 0.125. For each participant, a logistic curve was fit to endorsement rate at each eye-contact signal strength using Bayesian estimation (psignifit 4; Schütt et al., 2016), separately for forward and deviated faces. We used a 4-paramater psychometric function that estimates threshold (*m*), width (*w*), lapse rate (γ), and guess rate (γ). Because the task design forces participants to provide a response to each trial, the lapse rate and guess rate are generally zero. However, because chance performance and button press errors were still possible albeit rare, we accounted for these by fixing the lapse rate and guess rate at 2 percent to improve curve fit (Kingdom and Prins, 2010). As such, the 4-parameter equation essentially became a 2-parameter equation, with γ and λ being constants:

$$\psi\left( x \right|m,w)=\gamma+(1- \gamma-\lambda) \frac{1}{1+e^{-2\log\left( \frac{1}{.05} - 1 \right) \frac{x-m}{w}}}$$

where $\gamma=0.02, \lambda=0.02$

Although we estimated both the width (*w*) and threshold (*m*) parameters, for the present study, we were only interested in the width parameter (*w*). This is because width indexes perceptual precision, measuring sensitivity to small deviations in eye gaze direction across trials. As such, width primarily reflects low-level, sensory-based encoding of gaze information. Since our present study aims to isolate an intermediate perceptual stage linking basic visual processing with downstream social cognitive outcomes, we focused on width as the most appropriate measure of gaze perception. Conversely, threshold reflects the point at which gaze is judged as self-directed and has been shown to index self-referential bias, or the tendency to endorse eye contact. This judgment relies on higher-order cognitive processes, including prior knowledge, expectations, and beliefs about social intent, rather than sensory encoding of gaze direction.

After estimating the width parameter (in units of eye-contact signal strength) separately for deviated and forward faces, the values were converted back to units of gaze angle. To obtain a single measure to index perceptual precision during gaze perception, the measures were averaged across the two head orientation conditions.

- 1. *Gaze Task Eye Tracking Procedure*

For each face stimulus, an area of interest around the eyes was manually delineated (See Figure S1). Dwell time, or the average length of time spent fixating, on the eye area of interest prior to making a response was computed and used as a covariate in subsequent statistical analyses.

Prior to computing dwell time, several data processing procedures took place to ensure data validity. First, drift correction was performed for each trial by adjusting the post-stimulus eye position using the mean eye position during the 150 ms fixation period preceding stimulus onset. Next, eye positions contained by saccades and blinks (identified using the automated Eyelink algorithm) were discarded. Finally, trials containing blinks or saccades during the 150 ms pre-stimulus period were excluded, as accurate drift correction could not be performed.

**Figure S1. Example gaze perception stimulus with eye area of interest (AOI).** The red box indicates the manually defined AOI encompassing the eye region, which was used to compute dwell time (average fixation duration within the eye region prior to response) during the gaze perception task.


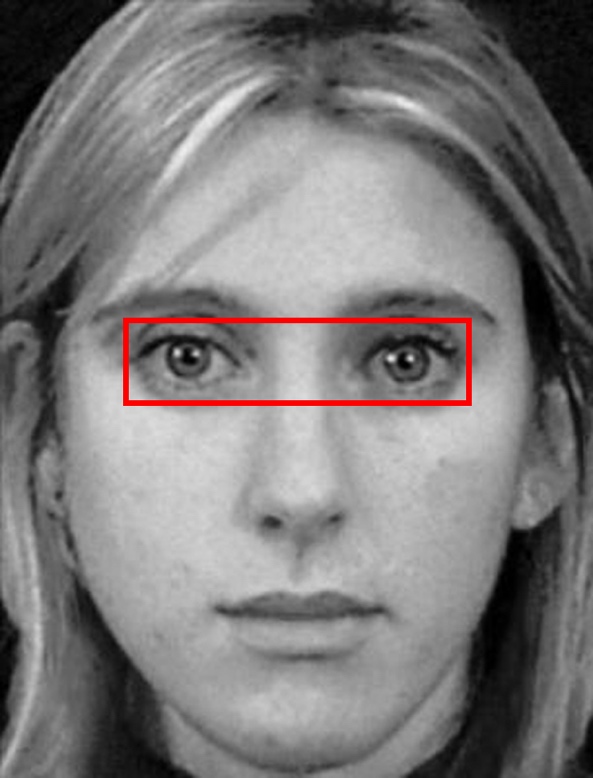


1. **Supplementary Analysis: Results of Pearson Correlations**

*Table S1: Inter-correlations between visual processing, gaze perception, mid- and higher-level social cognition variables.*

| **Variable** | **Correlations** | | | | | | | | | |
| --- | --- | --- | --- | --- | --- | --- | --- | --- | --- | --- |
|  | **1.** | | **2.** | | **3.** | | **4.** | | **5.** | |
| **1. Contrast Sensitivity** | **—** |  | |  | |  | |  | |  |
| **2. Visual Integration** | **0.09** | **—** | |  | |  | |  | |  |
| **3. Gaze Perception** | **0.26**** | **0.34***** | | **—** | |  | |  | |  |
| **4. Emotion Recognition** | **0.11** | **-0.09** | | **0.33***** | | **—** | |  | |  |
| **5. Theory of Mind** | **0.12** | **0.01** | | **0.12** | | **0.24**** | | **—** | |  |

*Note. ***p<.001, **p<.01, *p<.05.* Gaze perception was indexed by psychophysical width, where higher values indicate worse precision; thus, original correlation coefficients for correlations involving gaze perception were negative for visual integration (*r* = −0.34), for emotion recognition (*r*= −0.33), and for the theory of mind path (*r* = −0.12).

**3 Supplementary Analysis: Comparisons with Alternative Models**

To test the necessity and directionality of the path linking theory of mind and emotion recognition, we evaluated two alternative models. Model B, a more constrained model in which gaze perception predicted both emotion recognition and theory of mind directly, fit the data adequately (χ²(4) = 5.14, p = 0.27; CFI = 0.98; TLI = 0.95; RMSEA = 0.044). It did not improve upon Model A (Δχ² = 0.20, Δdf = 1, p = 0.65). Notably, the direct path from gaze perception to theory of mind was not significant in Model B, despite being freed to estimate. Critically, Model C, which reversed the positions of emotion recognition and theory of mind (i.e., basic visual processing → gaze perception→ theory of mind→ emotion recognition), demonstrated poor fit (χ²(5) = 18.98, p = 0.002; CFI = 0.71; TLI = 0.47; RMSEA = 0.137; Δχ² = 13.64, Δdf = 1, p < .001). Based on parsimony and model fit, we selected Model A, which assumes sequential propagation of effects through emotion recognition, over Models B and C.


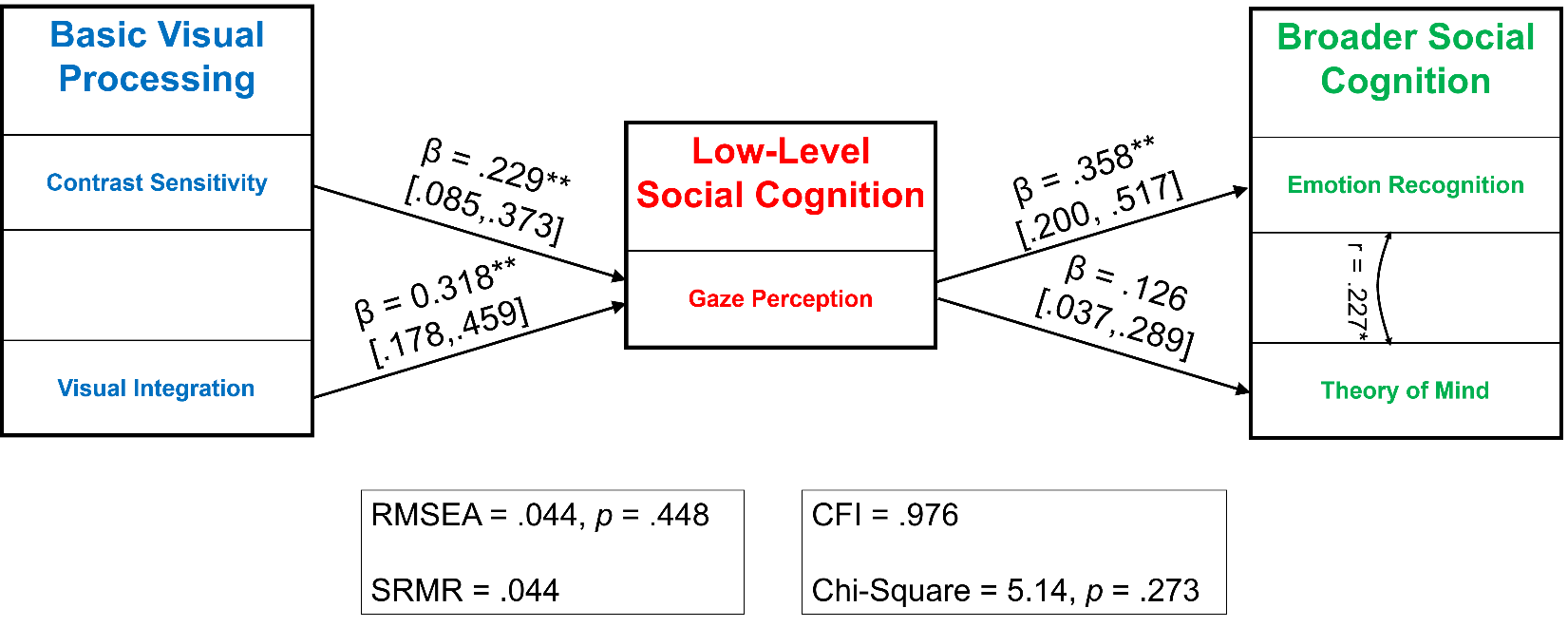


**Figure S2: Alternative Path** **model of** **gaze perception as the link between basic visual processing and broader social cognition (Model B)**. Model B demonstrated adequate fit to the data. However, gaze perception (in red) did not significantly predict theory of mind (in purple).

Note. ***p<.001, **p<.01, *p<.05. All path coefficients are reported as absolute values representing associations between better performance on each measure. Gaze perception was indexed by psychophysical width, where higher values indicate worse precision; thus, original coefficients for paths involving gaze perception were negative for visual integration (β = −0.32), for the gaze-to-emotion recognition path (β = −0.36), and for the gaze-to-theory of mind path (β = −0.13).

**Figure S3: Alternate Path** **model of** **gaze perception as the link between basic visual processing and broader social cognition (Model C)**. Model C demonstrated poor fit to the data. Gaze perception (in red) did not significantly predict theory of mind (in purple), but theory of mind (in purple) did significantly predict emotion recognition (in green).

Note. ***p<.001, **p<.01, *p<.05. All path coefficients are reported as absolute values representing associations between better performance on each measure. Gaze perception was indexed by psychophysical width, where higher values indicate worse precision; thus, original coefficients for paths involving gaze perception were negative for visual integration (β = −0.32) and for the gaze-to-theory of mind path (β = −0.13).


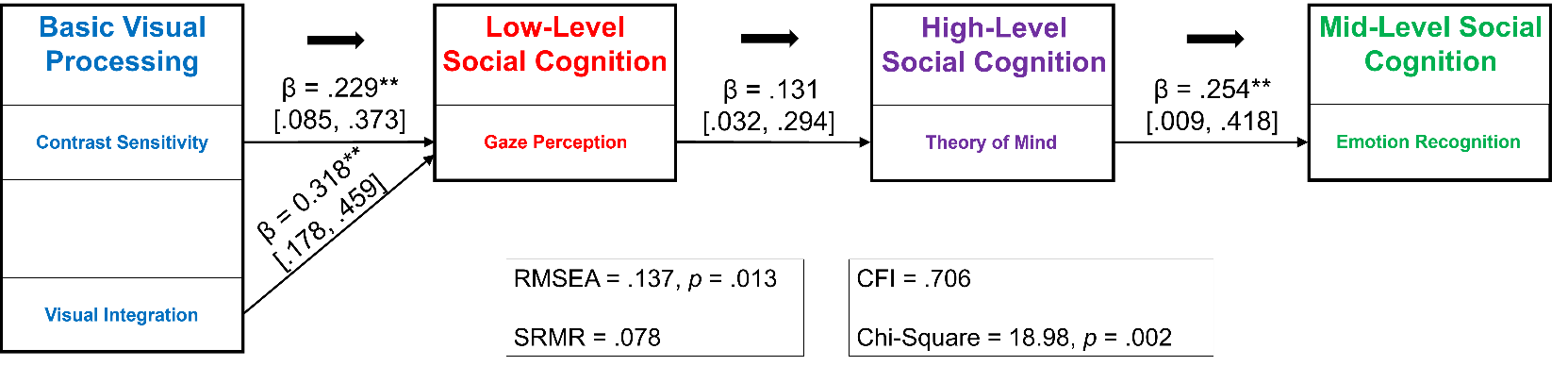


**4 Supplementary Analysis: Robustness to Demographic and Cognitive Covariates**

To ensure the robustness of our main findings, we conducted hierarchical regression analyses controlling for demographic variables (age, sex, socio-economic status), intellectual functioning (FSIQ), visual acuity, and eye dwell time during the gaze task. To do this, we used the ‘lm’ and ‘anova’ functions in R to run hierarchical regression analysis, where covariates were entered in the first block and predictors of interest were added in the second block.

First, we examined whether variance in gaze perception attributed to basic visual processing could be accounted for by demographic and cognitive covariates. In Model 1, demographic variables, FSIQ, visual acuity, and dwell time were entered. Contrast sensitivity and visual integration were then added in Model 2 to assess their unique contribution to gaze perception. Both contrast sensitivity (β = 0.26, p = 0.005) and visual integration (β = 0.22, p < 0.001) significantly predicted gaze perception, collectively explaining additional variance beyond the covariates (ΔR² = 0.122, p < 0.001; Table S2.1).

Next, we tested whether the association between gaze perception and emotion recognition remained significant after accounting for relevant covariates. Model 1 included demographic variables, FSIQ, and visual acuity. Gaze perception was entered in Model 2. Gaze perception significantly predicted emotion recognition (β = 0.31, p < 0.001), accounting for additional variance beyond the covariates (ΔR² = 0.080, p < 0.001; Table S2.2).

Finally, we assessed whether emotion recognition explained variance in theory of mind beyond demographic and cognitive factors. Demographic variables, FSIQ, and visual acuity were entered in Model 1, followed by emotion recognition in Model 2. Emotion recognition was a significant predictor of theory of mind performance (β = 0.23, p = 0.013), explaining additional variance over and above the covariates (ΔR² = 0.043, p = 0.013; Table S2.3).

*Table S2.1: Basic Visual Processes as Predictors of Gaze Perception*

| Model | Model Change Statistics | | | | Predictor Statistics | | |  |  |
| --- | --- | --- | --- | --- | --- | --- | --- | --- | --- |
|  | *df* | R^2^(ΔR^2^) | F | *p* | β | B | *p* |  |  |
| 1. Basic Visual Processes as Predictors of Gaze Perception | | | | | | | | |  |
| *Model 1* | 137 | 0.098(--) | -- | *--* |  |  |  | |  |
| (Intercept) |  |  |  |  | -- | 30.37 | <0.001 | |  |
| Sex |  |  |  |  | -0.17 | -1.71 | 0.043 | |  |
| Age |  |  |  |  | -0.13 | -0.15 | 0.099 | |  |
| SES Score |  |  |  |  | -0.10 | -0.49 | 0.258 | |  |
| FSIQ |  |  |  |  | -0.26 | -0.10 | 0.002 | |  |
| Visual Acuity |  |  |  |  | -0.16 | -1.41 | 0.444 | |  |
| Eye Dwell Time |  |  |  |  | 0.03 | 0.001 | 0.760 | |  |
| *Model 2* | 135 | 0.240(0.142) | 13.75 | <0.001 |  |  |  | |  |
| (Intercept) |  |  |  |  | -- | 36.48 | <0.001 | |  |
| Sex |  |  |  |  | 0.26 | -1.47 | 0.061 | |  |
| Age |  |  |  |  | -0.04 | -0.10 | 0.224 | |  |
| SES Score |  |  |  |  | -0.12 | -0.61 | 0.127 | |  |
| FSIQ |  |  |  |  | -0.29 | -0.09 | 0.002 | |  |
| Visual Acuity |  |  |  |  | -0.05 | -1.01 | 0.145 | |  |
| Eye Dwell Time |  |  |  |  | -0.04 | 0.002 | 0.434 | |  |
| Contrast Sensitivity |  |  |  |  | 0.26 | 8.09 | 0.005 | |  |
| Visual Integration |  |  |  |  | 0.22 | -19.17 | <0.001 | |  |
| Note. Gaze perception was indexed by psychophysical width, where higher values indicate worse precision; thus, the original regression coefficient for visual integration was negative (β = −0.22). | | | | | | | | |  |

*Table S2.2: Gaze Perception as a predictor of Emotion Recognition*

| Model | Model Change Statistics | | | | | | | | | Predictor Statistics | | | | |  |
| --- | --- | --- | --- | --- | --- | --- | --- | --- | --- | --- | --- | --- | --- | --- | --- |
|  | *df* | R^2^(ΔR^2^) | | F | | | *p* | | β | | B | | | *p* |  |
| 1. Gaze Perception as a Predictor of Emotion Recognition | | | | | | | | | | | | | | | |
| *Model 1* | 125 | | 0.033(--) | | -- | *--* | |  | | | |  |  | | |
| (Intercept) |  | |  | |  |  | | -- | | | | 21.58 | <0.001 | | |
| Sex |  | |  | |  |  | | 0.14 | | | | 1.14 | 0.110 | | |
| Age |  | |  | |  |  | | 0.01 | | | | 0.01 | 0.901 | | |
| SES Score |  | |  | |  |  | | -010 | | | | -0.40 | 0.260 | | |
| FSIQ |  | |  | |  |  | | 0.13 | | | | 0.04 | 0.144 | | |
| Visual Acuity |  | |  | |  |  | | 0.21 | | | | 1.37 | 0.023 | | |
| *Model 2* | 124 | | 0.113(0.080) | | 12.23 | <0.001 | |  | | | |  |  | | |
| (Intercept) |  | |  | |  |  | | -- | | | | 29.04 | <0.001 | | |
| Sex |  | |  | |  |  | | 0.10 | | | | 0.78 | 0.256 | | |
| Age |  | |  | |  |  | | 0.01 | | | | -0.01 | 0.835 | | |
| SES Score |  | |  | |  |  | | -0.14 | | | | -0.53 | 0.119 | | |
| FSIQ |  | |  | |  |  | | 0.04 | | | | 0.01 | 0.672 | | |
| Visual Acuity |  | |  | |  |  | | 0.17 | | | | 1.12 | 0.053 | | |
| Gaze Perception |  | |  | |  |  | | 0.31 | | | | -0.26 | <0.001 | | |
| Note. Gaze perception was indexed by psychophysical width, where higher values indicate worse precision; thus, the original regression coefficient for gaze perception was negative (β = −0.31). | | | | | | | | | | | | | | | |

*Table S2.3: Emotion Recognition as a predictor of Theory of Mind*

| Model | Model Change Statistics | | | | | | | | | Predictor Statistics | | | | |  |
| --- | --- | --- | --- | --- | --- | --- | --- | --- | --- | --- | --- | --- | --- | --- | --- |
|  | *df* | R^2^(ΔR^2^) | | F | | | *p* | | β | | B | | | *p* |  |
| 1. Emotion Recognition as a predictor of Theory of Mind | | | | | | | | | | | | | | | |
| *Model 1* | 119 | | 0.004(--) | | -- | *--* | |  | | | |  |  | | |
| (Intercept) |  | |  | |  |  | | -- | | | | 0.72 | <0.001 | | |
| Sex |  | |  | |  |  | | 0.04 | | | | 0.01 | 0.643 | | |
| Age |  | |  | |  |  | | -0.06 | | | | -0.001 | 0.503 | | |
| SES Score |  | |  | |  |  | | -010 | | | | -0.01 | 0.279 | | |
| FSIQ |  | |  | |  |  | | 0.14 | | | | 0.001 | 0.113 | | |
| Visual Acuity |  | |  | |  |  | | 0.01 | | | | 0.003 | 0.875 | | |
| *Model 2* | 124 | | 0.047(0.043) | | 6.34 | 0.013 | |  | | | |  |  | | |
| (Intercept) |  | |  | |  |  | | -- | | | | 0.57 | <0.001 | | |
| Sex |  | |  | |  |  | | 0.02 | | | | 0.004 | 0.256 | | |
| Age |  | |  | |  |  | | -0.06 | | | | -0.001 | 0.835 | | |
| SES Score |  | |  | |  |  | | -0.08 | | | | -0.009 | 0.119 | | |
| FSIQ |  | |  | |  |  | | 0.12 | | | | 0.001 | 0.672 | | |
| Visual Acuity |  | |  | |  |  | | -0.03 | | | | -0.005 | 0.053 | | |
| Emotion Recognition |  | |  | |  |  | | 0.23 | | | | 0.01 | 0.013 | | |
|  | | | | | | | | | | | | | | | |

**References**

Kingdom, F.A.A., Prins, N., 2010. Psychophysics: A practical introduction. Elsevier Academic Press, San Diego, CA, US.

Muthén, L.K. and Muthén, B.O. (1998-2017). Mplus User’s Guide. Eighth Edition. Los Angeles, CA

Schütt, H. H., Harmeling, S., Macke, J. H., & Wichmann, F. A. (2016). Painfree and accurate Bayesian estimation of psychometric functions for (potentially) overdispersed data. *Vision research*, *122*, 105-123. doi: 10.1016/j.visres.2016.02.002
